# Knowledge, illness perceptions and stated clinical practice behaviour in management of gout: a mixed methods study in general practice

**DOI:** 10.1007/s10067-016-3212-2

**Published:** 2016-02-22

**Authors:** Bart Spaetgens, Tobias Pustjens, Lieke E. J. M. Scheepers, Hein J. E. M. Janssens, Sjef van der Linden, Annelies Boonen

**Affiliations:** 1 Department of Internal Medicine, Division of Rheumatology, Maastricht University Medical Centre, P.O. Box 5800, 6202 AZ Maastricht, The Netherlands; 2 CAPHRI Research Institute, University Maastricht, Maastricht, The Netherlands; 3 Department of Primary and Community Care, Radboud University Medical Centre, Nijmegen, The Netherlands; 4 Primary Health Care Centre Lobede, Lobith-Tolkamer, The Netherlands

**Keywords:** Attitudes, Gout Knowledge Questionnaire, Health beliefs, Illness perceptions, Primary care

## Abstract

The objective of the present study is to explore knowledge, illness perceptions and stated practice behaviour in relation to gout in primary care. This is a mixed methods study among 32 general practitioners (GPs). The quantitative assessment included the Gout Knowledge Questionnaire (GKQ; range 0–10; better) and Brief Illness Perceptions Questionnaire (BIPQ; nine items, range 0–10; stronger). Structured individual interviews obtained further qualitative insight into knowledge and perceptions, in the context of daily practice. Among 32 GPs, 18 (56.3 %) were male, mean age 44.4 years (SD 9.6) and mean working experience 17.1 years (SD 9.7). Median score [interquartile ranges (IQR)] on the GKQ was 7.8 [6.7–8.9] and 9.0 [8.0–10.0], when presented as open or multiple-choice questions, respectively. The BIPQ (median; [IQR]) revealed that gout was seen as a chronic disease (8.0; [7.0–9.0]), affecting life and emotions moderately (6.5; [5.0–7.0]), having many severe symptoms (8.0; [7.0–9.0]) and in which treatment could be very helpful (8.0; [7.0–9.0]). Further interviews revealed large variation in specific aspects of knowledge and about gaps concerning indications for uric acid-lowering therapy (UALT), duration of UALT, target serum uric acid (sUA) level or duration of prophylactic treatment. Finally, patients’ adherence was not checked systematically. Specific knowledge gaps and discrepancies between perceptions and stated practice behaviour were identified, which might hamper effective management of this well-treatable disease. Improving evidence on the rationale and effectiveness of treatment targets and adherence interventions, tailoring guidelines to general practice and intensification of implementation of guidelines in primary health care seem to be needed.

## Introduction

Gout is a chronic rheumatic disease with a reported prevalence of 2.5 % in the UK and 3.9 % in the USA, making it the most common inflammatory joint disease [1–3]. Despite being a well-treatable disease, it is recognized that the management of gout is suboptimal in both primary [4] and secondary care [5]. In a primary care study among patients with gout, low levels of allopurinol prescribing (57 %), serum uric acid (sUA) level testing (55 %) and achievement of target sUA level (<0.36 mmol/L) (22.4 %) during a 5-year study period were shown [6]. In secondary care, adherence to American College of Rheumatology (ACR) guideline recommendations by rheumatologists could be highly improved, shown by a mean adherence score of 5.8 out of 8 ACR guideline recommendations. Low adherence on first-line uric acid-lowering therapy (UALT) dosage, acute prophylaxis dosage and length of prophylaxis was shown [5]. Common barriers for effective management can be distinguished into patient and physician barriers. Important barriers among patients were not only misperception of the severity and chronicity of gout, but also inadequate patient education resources, resulting in poor adherence to treatment [7–9]. Among physicians, underestimation of long-term complications and insufficient knowledge about the indications for UALT and about adequate dosing of UALT have already been suggested [10, 11].

On the same line, some qualitative studies explored barriers to effective gout management among patients and physicians [12–16]. Despite general practitioners (GPs) being the most relevant (in most countries) health care professionals when it comes to diagnosing and treating the disease, the studies in current literature included only a low number of GPs. Therefore, broad insight into how gout is managed by GPs is missing. Notwithstanding, linking knowledge, perceptions and stated practice behaviour is essential when planning to improve gout management for patients with gout.

The current study uses a quantitatively and qualitatively approach to understand knowledge, illness perceptions and stated clinical behaviour of GPs when managing gout with specific attention to the role of UALT, sUA level and prophylactic treatment.

## Materials and methods

### Study design and data collection

A mixed methods approach was used to investigate and understand specific knowledge gaps in pathophysiology and management of gout, illness perceptions about the disease and clinical stated practice behaviours in management. The Good Reporting of A Mixed Methods Study (GRAMMS) guidelines as provided by the Enhancing the QUAlity and Transparency Of health Research (EQUATOR) network were followed [17, 18].

The study was conducted in the southern part of the Netherlands. During a period of 2 weeks, GPs were asked to participate in the study. After agreeing, questionnaires on demographics, gout knowledge and gout perceptions were administered, followed by a structured interview to explore in-depth understanding and to relate both issues to practice behaviour (TP). The structured interviews were audiotaped and transcribed verbatim. The GPs consented to quote part of the interviews in anonymized form.

### Demographics

General questions about age, sex, years working experience as GP, hours involved in patient care, estimated number of patients with gout per year, practice type, recent education on gout (within the last year, yes/no) and familiarity with gout (on a 0–10 scale, 0 being not familiar and 10 being extremely familiar) were recorded.

### Gout Knowledge Questionnaire

The Gout Knowledge Questionnaire (GKQ) aims to assess knowledge of patients or physicians and addresses ten multiple-choice questions related to the pathogenesis, treatment of acute attacks and also management of chronic gout [19, 20]. GPs were first asked to answer the questions while blind for the answer options. In case that they hesitated (or did not use one of the questionnaire answer options), the interviewer showed the original GKQ multiple-choice answers. The GKQ was previously translated into Dutch according to the International Society for Pharmacoeconomics and Outcome Research (ISPOR) principles of good practice [21], which is consistent with the approach proposed as best practice in rheumatology by Beaton [22]. As each correct answer provides a score of one, the total score ranges from 0 to 10, with higher scores indicating better knowledge.

### Brief Illness Perceptions Questionnaire

When completing the validated Dutch version of the Brief Illness Perceptions Questionnaire (BIPQ) [23, 24], the GPs were asked to rate their personal perceptions, while imagining that they would suffer from gout with an “average” disease course. The BIPQ is a nine-item questionnaire that assesses cognitive and emotional perceptions of a disease, within nine domains (Q1: consequences, Q2: timeline, Q3: personal control, Q4: treatment control, Q5: identity, Q6: coherence, Q7: emotional representation, Q8: concern, Q9: cause). Q1 to Q8 are scored on an 11-point numeric rating scale (0 to 10), with higher scores representing a more threatening view per domain. Q9 additionally permits to list up to three items that play a causative role in the disease.

### Structured interviews

After completing the questionnaires, the GPs were interviewed to gain more in-depth insight into knowledge and perceptions and link these to clinical practice behaviour, specifically with regard to the role of sUA in diagnosis and follow-up, appropriate usage of UALT and the role of adherence in relation to management of the disease.

### Data analysis

Descriptive statistics were used to present the demographics and results of questionnaires, and means with standard deviation (SD) or medians with interquartile ranges [IQR] were used depending on skewness of data.

For qualitative analysis, the verbatim transcripts were read repeatedly and independently by two readers (BS, LS). Using the grounded theory approach, a coding system with categories that were identified in the previous step was developed as well as a taxonomy of the data [25]. The two readers met regularly to discuss coding and interpretation of data. Wherever necessary, consensus was reached after discussing specific passages or a third reader (AB) acted as referee. Representative quotes were collected during data analysis and reported based on the frequency of the particular (and similar) quotes.

## Results

### Demographic characteristics

Thirty-two GPs were interviewed. Eighteen (56.3 %) were male; the mean age was 44.4 years (SD 9.6 years); the mean number of years of working experience as GP is 17.1 years (SD 9.7 years). The GPs were 34.1 h (SD 11.0) per week involved in patient care, and only four (12.9 %) had followed an educational event on gout in the past year. The estimated number (mean) of new patients with gout in their practice was 8.9 (SD 7.0) per year, and familiarity with the disease was scored as 7.0 out of 10.0 (SD 1.1) (Table 1).

**Table 1:** Baseline characteristics for general practitioners (GP) (*n* = 32)

Age (years), mean ± SD	44.4 ± 9.6
Male sex; *n* (%)	18 (56.3)
Practice type, *n* (%)	
Group practice	15 (46.9)
Private practice	4 (12.5)
Self-employed substitute	10 (31.2)
Other	3 (9.4)
Years’ experience as GP, mean ± SD	17.1 ± 9.7
Hours involved in patient care, mean ± SD [range]	34.1 ± 10.9 [8–55]
Estimated new patients with gout per year, mean (median), [IQR]	8.9 (7.0) [4.3–11.5]
Recent (<1 year) education in gout, *n* (%)	5 (15.6)
Self-reported gout familiarity (score 0–10), mean ± SD [range]	7.0 ± 1.1 [5–9]
Gout Knowledge Questionnaire (score 0–10), mean (median), [IQR]	
Open answers Multiple-choice answers	7.4 (7.8) [6.7–8.9] 9.1 (9.0) [8.0–10.0]

### Gout knowledge

The mean scores (number of correct answers) for the GKQ were 7.4 (median 7.8) [IQR 6.7–8.9] and 9.1 (9.0) [8.0–10.0] when answering an open or multiple-choice question with the original answer options, respectively. The numbers (%) of GPs with correct answers for each item are summarized in Table 2. Questions on the cause of gout (Q1, Q3), signs indicating an acute attack (Q2), treatment of an acute attack (Q4) and recognition of allopurinol being UALT (Q5) were correctly answered by 88 to100 % of the GPs in the open questioning part, respectively. Questions on flare prevention (Q9) and comorbidity (Q10) were answered correctly by 72 and 50 %, respectively, but improved to 97 and 100 % when presenting the original answer options. On the other hand, the question on the target value (“ideal value”) (Q6) was answered correctly by 12 %, in the open question, but it increased to 84 % when presenting the original answer options. Finally, the questions about non-pharmacological interventions (Q7) and duration of UALT (Q8) improved to only 75 and 69 % correct answers after seeing the answer options.

**Table 2:** Gout knowledge level of general practitioners per question (*n* = 32)

Question	Open question correct answered, *n* (%)	Multiple-choice correct answered, *n* (%)
1. Q: What causes gout? Answer options: (a) too little calcium, *(b) too much uric acid*, (c) an infection, (d) diabetes	31 (96.9)	32 (100)
2. Q: How do you know if you have an acute attack of gout? Answer options: *(a) you have a painful swollen joint*, (b) you have a change in blood tests, (c) your skin gets red and itchy, (d) you have a lump on your ear	30 (93.8)	32 (100)
3. Q: What inside the joint causes attacks of gout? Answer options: (a) bacteria, (b) viruses, *(c) crystals*, (d) calcium	30 (93.8)	32 (100)
4. Q: Which of these is a good treatment during a sudden painful attack of gout in someone with no other medical condition? Answer options: (a) exercise, (b) allopurinol, *(c) NSAIDs like ibuprofen, naproxen or indomethacin*, (d) benzbromarone	32 (100)	32 (100)
5. Q: Lowering your blood uric acid can help prevent future attacks of gout. Which of these drugs can lower your blood uric acid? Answer options: *(a) allopurinol*, (b) prednisone, (c) NSAIDs like ibuprofen, naproxen or indomethacin, (d) colchicine	28 (87.5)	32 (100)
6. Q: What is the ideal blood uric acid level to aim for after treatment of gout? Answer options: (a) lower than 0.59 mmol/L, (b) lower than 0.48 mmol/L, *(c) lower than 0.36 mmol/L*, (d) lower than 0.12 mmol/L	4 (12.5)	27 (84.4)
7. Q: In order to reduce the serum uric acid, what can you do in addition to medications? Answer options: (a) drink more beer, (b) eat more seafood, (c) eat more red meat, *(d) lose weight if you are overweight*	Not applicable	24 (75.0)
8. Q: If you are taking a drug to lower your blood uric acid levels, how long do you need to take this drug? Answer options: (a) 1 month, (b) 1 year, (c) 2 years, *(d) forever*	20 (62.5)	22 (68.8)
9. Q: When taking a drug to lower your blood uric acid levels, there can be a temporary increase in gouty attacks. How can you prevent such attacks? Answer options: (a) skip doses of the drug and restart, (b) drink less water, (c) drink alcohol every day, *(d) take daily colchicine*	23 (71.9)	31 (96.9)
10. Q: Which is a medical condition that is common in patients with gout? Answer options: *(a) high blood pressure*, (b) cancer, (c) AIDS, (d) asthma	16 (50.0)	30 (93.8)
Total correct score mean (median) [IQR]	7.4 (7.8) [6.7–8.9]	9.1 (9) [8–10]

Correct answers are in italic

### Illness perceptions about gout

The results of the perceptions of GPs about gout are summarized in Table 3. GPs considered gout to be a chronic disease (Q2: median 8.0), with (a considerable number of) severe symptoms (Q5: median 8.0), but with moderate impact on life and emotions (Q1 and Q8: median 6.5), and for which treatment is very helpful (Q4: median 8.0). They believed that gout is not strongly influenced by personal actions (Q3: median 4.0). A large variation was observed in perceptions of the amount of concerns gout can raise (Q6: median 5.0, IQR 3.3–6.8) and the level of understanding of the disease (median 6.0, IQR 3.3–7.0). Finally, 16 of 32 (50 %) reported diet (alcohol, obesity) to be a major contributing cause of gout (Q9).

**Table 3:** Results of the BIPQ in general practitioners

	Mean (median) [IQR]	*n* (%)
Q1 Consequences (10 = severely affects life)	6.2 (6.5) [5.0–7.0]	
Q2 Timeline (10 = continues forever)	7.5 (8.0) [7.0–9.0]	
Q3 Personal control (10 = extreme amount)	4.3 (4.0) [3.0–5.0]	
Q4 Treatment control (10 = extremely helpful)	7.8 (8.0) [7.0–9.0]	
Q5 Identity score (10 = many severe symptoms)	7.7 (8.0) [7.0–9.0]	
Q6 Illness concern (10 = extremely concerned)	5.0 (5.0) [3.3–6.8]	
Q7 Coherence (10 = understands very clearly)	5.7 (6.0) [3.3–7.0]	
Q8 Emotional representation (10 = extremely affected emotionally)	6.2 (6.5) [5.0–8.0]	
Q9 Top listed causes: 1. Diet (alcohol, obesity) 2. Hereditary 3. Medication (i.e. diuretics)		
		16 (50.0)
		13 (40.6)
		12 (37.5)

### Qualitative analysis on knowledge, beliefs and practice behaviour

Table 4 shows the most frequent quotes per topic that were collected during the data analysis.

**Table 4:** Themes from qualitative analysis with representative quotes

Number / Themes	Quotes
1. Knowledge	1. “I don’t know the target level of serum uric acid; I always look in the lab form for the reference values” (*which are 0.20–0.42 mmol/L*)
2. Illness perceptions	1. “Gout is a chronic devastating systemic disease, leading to functional disability”. 2. “The associated kidney disease or heart failure are very serious conditions, but the acute attacks are the worst for patients”.
3. Necessity of uric acid	1. “Gout cannot be diagnosed without the presence of hyperuricemia”. 2. “Serum uric acid is not useful, because it will be low in patients with an acute attack”
4. Treatment with UALT	1. “The main reason to start with UALT is when patients have more than 3 gout attacks per year”. 2. “If patients have fewer attacks (e.g. <3), but the complaints are very severe, then this is a reason to start UALT”.
5. Duration of treatment with UALT	1. “Allopurinol is prescribed lifelong, unless patients change their lives in such a way, you do not expect them to get gout attacks anymore (after weight reduction or stopping diuretics)”. 2. “If patients have no gout attacks for a longer period of time (e.g. 1 year), I try to reduce and thereafter stop the UALT”.
6. Flare prophylaxis	1. “I combine allopurinol and colchicine to prevent acute gout flares, for a period of 2–4 weeks”. 2. “I never prescribe allopurinol after an acute flare, first I prescribe colchicine (or a NSAID) and after 4 weeks I stop it and start allopurinol”. 3. “I do not prescribe prophylactic treatment, I advise patients to drink more and sometimes stop diuretics”.
7. Target level serum uric acid	1. “The target level of 0.36 mmol/L is not a strict treatment goal. I accept higher serum uric acid levels if the number of acute attacks is decreased”. 2. “If patients have gout, I try to reduce the serum uric acid level below 0.36 mmol/L in order to reduce the hyperuricemia-associated risk of cardiovascular events. Furthermore, I will check and if necessary adjust cholesterol, blood pressure and glucose”.
8. Adherence	1. “Adherence to UALT is not a problem in patients with gout, since they are well aware of the fact they will get new gout attacks if they do not take their medication”. 2. “I think patients with gout take their medication (UALT) very well in the beginning, but in the course of time become less adherent. Then these patients will return with a gout flare”. 3. “I have too little time to check whether patients with gout are adherent”.
9. Lifestyle advices	1. “I refer my patients to a website (www.thuisarts.nl)^a^ where al truths and untruths about gout are presented. If I am correct, there is no evidence for all these dietary advices” 2. “I give the same lifestyle advices as I give patients in cardiovascular risk management” 3. “I warn patients for the possible danger of alcohol and organ meats. Also, I try to motivate them to lose some weight”

^a^A Dutch website with the most essential information in plain language, understandable by patients, about diseases treated by GPs, an initiative from the Dutch College of GPs

### Assessment of serum uric acid and (use) of uric acid lowering therapy

First, divergent opinions about the usefulness of sUA to diagnose gout were observed. Ten GPs believe that sUA is necessary to diagnose gout, as gout cannot be diagnosed in the absence of hyperuricaemia. Twelve GPs indicated that sUA levels are required in some specific situations, namely the following: (1) “to differentiate gout from other diagnoses in atypical cases” (six GPs) and (2) “to strengthen the diagnosis of gout, which will lead to better treatment” (six GPs). The remaining ten GPs felt that sUA is not necessary and not even useful to diagnose gout. Reasons were (1) “gout is a clinical diagnosis, preferably confirmed by joint aspiration” (four GPs) and 2) “a low sUA level does not exclude gout” (three GPs) and “sUA may be low, in particular when patients have an acute gouty arthritis” (three GPs).

Second, reasons to start treatment with UALT were very diverse. The most important reasons were the number of gout attacks per year: “The main reason to start with UALT is when patients have more than 3 gout attacks per year”, severity of symptoms: “If patients have fewer attacks (e.g. <3), but the complaints during the attack are severe, then this is a reason to start UALT” and hyperuricaemia in case of a gout attack. Only six GPs mentioned tophi as reason to start with UALT, and three of these GPs determined the effectiveness of UALT, based on the resolution of (if present) tophi.

Third, with regard to duration of UALT, 12 GPs did not prescribe lifelong UALT, for one or more different reasons. Seven of these GPs tried to stop the UALT after 1 year: “If patients have no gout attacks for a longer period of time (e.g. 1 year), I try to reduce and eventually stop UALT”; five GPs suggested that UALT could be stopped after adjustment of lifestyle: “Allopurinol is prescribed lifelong, unless patients change their lives in such a way, you do not expect them to get gout attacks anymore (after weight reduction or stopping diuretics)”; six GPs terminated UALT in the occurrence of renal impairment. One GP thought that allopurinol could be used to treat an acute gouty arthritis.

Fourth, when initiating UALT, nine did not add prophylactic treatment to prevent flares. These GPs advised changing medication/lifestyle (three GPs), prescribed higher doses (or a combination) of UALT in case of flares during the drug start-up phase (three GPs) or waited until the patient was attack-free for a longer period before starting UALT (three GPs). Of the 23 GPs starting colchicine or a non-steroidal anti-inflammatory drug (NSAID) during UALT start-up, none prescribed prophylactic treatment for longer than 2 months: “I combine allopurinol and colchicine to prevent acute gout flares, for a period of 2–4 weeks”. (14 GPs).

Finally, to determine effectiveness of UALT, 26 GPs determined sUA, of which six only in case patients continue to have attacks. Seventeen GPs explicitly stated that they did not strive for the target level of 0.36 mmol/L but based effectiveness of UALT on the absence of new gout attacks and stated that higher sUA levels were acceptable: “The target level of 0.36 mmol/L is not a strict treatment goal. I accept higher serum uric acid levels if the number of acute attacks is decreased”. Six GPs never determine sUA to monitor treatment.

### Adherence to drug therapy

Nineteen GPs believed that patients with gout are adherent to their drug treatment. “Patients are well aware of the fact new gout attacks will occur if they don’t take their medication”. Of the 13 GPs that assumed that patients were not adherent to therapy, nine GPs believed that they were adherent in the beginning but stop UALT over time: “I think patients with gout take their medication (UALT) very well in the beginning, but in over the course of time become less adherent”. All GPs assumed that these patients would restart therapy themselves in case of a new attack

Only eight GPs actively monitored patient adherence by planning appointments at a regular interval (varying from 1 month in the start-up phase to once a year), during which two determined sUA to assess adherence. Seven GPs check adherence when patients had an appointment for any reason. Furthermore, when specifically inquired, 12 confirmed that they checked regularly whether patients pickup their repeat prescriptions, but only electronically and no contact with the non-adherent patients would follow. If non-adherence was recognized (in any way), only 12 GPs would discuss the effects and complications of being non-adherent. Ten GPs admitted to spend insufficient effort in the follow-up of adherence. Main reasons are lack of time or beliefs that patients are adherent anyhow.

### Lifestyle advice in patients with gout

Sixteen GPs believed that diet and drinking habits were main contributing causes of gout (BIPQ (Q9)), and all of these mentioned that adjustment of these factors (weight loss, less alcohol, no organ meats, drink more water) would lower sUA in addition to medication. It was therefore surprising to see that only four GPs gave any lifestyle advice(s) to patients with gout. Seven GPs explicitly mentioned that adjustment of diet was outdated.

## Discussion

**Fig. 1:**
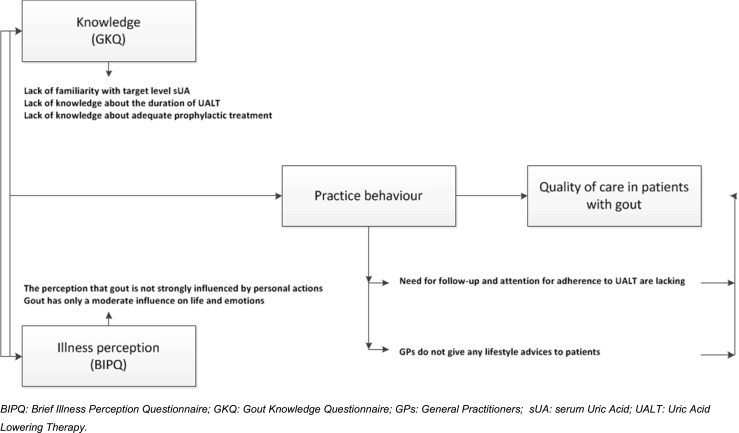
Identified barriers to optimal management in patients with gout treated by general practitioners

Our study adds fuel to the ongoing debate about why gout, a treatable disease, is often insufficiently controlled [8, 26]. The strength of this study is that it is the first to address, at the same time, knowledge, illness perceptions and stated clinical practice behaviour in GPs, the medical professionals that commonly diagnose and treat gout. Moreover, the use of a mixed quantitative and qualitative approach allowed to gain in-depth insight into the consequences of gout knowledge and (inadequate) perceptions on gout in general practice, while, at the same time, providing an overall quantification. In Fig. 1, we summarized several potential barriers identified in our study and illustrated graphically how these barriers might eventually effect quality of care in patients with gout.

The GPs’ knowledge, as measured with the GKQ, on pathophysiology, signs and symptoms and treatment of an acute gout attack was mostly excellent, although only half of them indicated that dietary factors play a causative role in gout. However, GKQ-data combined with interviews on knowledge and practice behaviour learned that there is a large variation in the *long-term* management of gout, specifically in reasons to start UALT, the duration of UALT prescribing and prophylactic treatment at initiation of UALT. The latter finding is in line with one other study already showing inappropriate use of prophylactic colchicine among 74 % of the patients under care of a primary care physician [27]. Furthermore, although GPs have a pragmatic and realistic view on the evaluation of effectiveness of UALT, it was interesting that most GPs were not aware of the sUA target level of 0.36 mmol/L as recommended by guidelines and stated to use (if any) the upper limit of laboratory normal ranges (0.42 mmol/L). Finally, half of the GPs indicated that dietary factors play a causative role in gout, but only few would give lifestyle advices to improve and eliminate causative factors, although it might be attributed partly due to lack of high-quality evidence for specific dietary interventions (avoidance of alcohol, weight loss) [28]. Moreover, lifestyle interventions could have a role in management of gout-associated comorbidities (e.g. cardiovascular diseases, renal disease).

It is well known that also perception of the burden of disease influences the dedication of professionals to a disease and its management. It was therefore reassuring that the GPs perceived gout as a chronic disease with severe symptoms and important impact, in which treatment is very helpful. The GPs’ illness perceptions are in accordance with those of 142 patients with gout, which showed that patients also viewed gout as a chronic condition responsive to therapy, but not influenced by personal actions [29]. Nevertheless, there was a striking unawareness among GPs with respect to need for follow-up and/or attention for adherence, as most GPs sincerely believed that patients were adherent to treatment. Other reasons to not make follow-up appointments or check adherence regularly were lack of time or believe that patients who were non-adherent would present themselves automatically when having a new gout flare, actually referring to the patients’ personal responsibility. So, even when adherence was checked by the GP, actions to improve inadequate adherence were rare.

We realize that the interpretation of the results of our study might be difficult, since we did not actually evaluated quality of care by auditing GPs’ adherence to treatment guidelines or quality indicators (QI). Nevertheless, using the ACR and European League Against Rheumatism (EULAR) guidelines as external standard [30–32], we implicitly took a large number of the formulated QI by Mikuls et al. [33] into account. A first example would be the QI about the role of follow-up of sUA level when prescribing UALT: “IF a gout patient is given a prescription for a xanthine oxidase inhibitor, THEN a serum urate level should be checked at *least once* during the first 6 months of continued use, BECAUSE periodic serum urate measurements are required for appropriate dose adjustments of xanthine oxidase inhibitors (escalations or reductions)”. A second example would be the QI about behavioural modifications: “IF a patient is diagnosed with gout and has *either* (1) obesity (defined as a body mass index ≥28 kg/m^2^) or (2) frequent alcohol use (≥1 alcoholic beverage per day), THEN as part of their overall therapy, patients should be advised on the importance of weight loss *and/or* decreased alcohol use, BECAUSE weight loss and reduction of alcohol intake may be beneficial components of gout therapy”. On this line, it is important to realize that the guideline on “arthritis”, including recommendations how to diagnose and manage gout of the Dutch College of General Practitioners (NHG), currently does not mention a specific sUA level as a treatment target, does not recommend prophylactic treatment when initiating UALT and does not provide specific advises on behavioural modifications, follow-up or monitoring of adherence for patients with gout [34]. On the other hand, while the GPs’ standard mentioned the presence of tophi as indication to initiate UALT, 26 GPs do not mention tophi as a reason to start UALT.

Although guidelines are a good starting point to improve quality of care, it is well known that recommendations do not guarantee ubiquitous agreement or compliance with them. Harrold et al. reported among a random sample of US PCPs (including 444 GPs) that only 9.6 % of the GPs were aware of the guidelines and adhered to recommended treatment for acute, intercritical and tophaceous gout in only 47, 3.4 and 12.5 % of the cases, respectively [35]. In addition to (non)awareness, physicians (including GPs) are experts with strong opinions whom might not always agree with recommendations in guidelines and might question the evidence. Although already a large amount of evidence is available and summarized in the 2006 EULAR and 2012 ACR guidelines, it should be recognized that the strength of evidence for several recommendations, such as the role and value using sUA as a target for treatment, still needs improvement. As such, we believe that QI that are part of GP’s audit might be more effective. Nevertheless, these QI still require strong evidence and a costly organization for monitoring and auditing.

Last, the differences in views between GP and international guidelines might be explained by the heterogeneity of the disease itself and important differences in disease spectra between primary and secondary care will be present. Undoubtedly, GPs treat the milder cases. Therefore, one of the outstanding issues is to collect high-quality registry data in primary care and identify factors that might predict poor prognosis.

This study has other limitations that need to be addressed. First, GPs were recruited from one region in the Netherlands. This might limit the generalizability of these results to all GPs in (and outside) the Netherlands. Nevertheless, we included a broad spectrum of GP that was also representative for the Dutch situation, with regard to years of working experience, sex distribution and age, as this is necessary for qualitative studies (in the Netherlands, 56 % of the GPs are male with a mean age of 48.8 years and of which 46 % have a fulltime employment). As such, the current study represents to date the largest qualitative study in gout. The number of 32 GPs is acceptable from a quantitative view, as for the qualitative part of the study, the theoretical saturation points of information were reached. Second, the GKQ was developed as a multiple-choice questionnaire with some of the multiple-choice answers being too obvious in our opinion. Therefore, the questionnaire was presented first with open-ended items (i.e. hiding the answer options), thereby eliciting quotes and thus supporting the qualitative analyses. Finally, in our study, as in any study with qualitative analyses, it might be possible that the interviewer, the questionnaires (that were completed before the interview) or the semi-structured character of the interview itself unintentionally influenced the GPs’ answers.

In conclusion, among a sizable proportion of GPs, we have identified specific knowledge gaps and discrepancies between illness perceptions and stated clinical practice behaviour of GPs that might imply risks for shortcoming patient management in primary health care. Improvement of knowledge of evidence-based treatment targets, implementing adherence interventions and tailoring up-to-date guidelines to general practice are needed to ultimately improve the care of all patients with gout.
